# Formulation and In Vitro Evaluation of Ufasomes for Dermal Administration of Methotrexate

**DOI:** 10.5402/2012/873653

**Published:** 2012-06-12

**Authors:** Arvind Sharma, Sandeep Arora

**Affiliations:** Chitkara College of Pharmacy, Chitkara University, Punjab, India

## Abstract

Dermal drug delivery system that is required to localizes methotrexate (MTX) in the synovial joint is needed to treat inflammation in rheumatoid arthritis (RA). The present investigation aims at exploring the potential of fatty acid vesicles for the topical delivery of methotrexate. Vesicles were prepared by film hydration method using oleic acid as a fatty acid principal component. Developed vesicles were characterized for size, size distribution, shape, in vitro release, pH dependent, and storage stability. Interaction between MTX and oleic acid was investigated using differential scanning calorimetry. The MTX amount permeated through rat skin was three- to fourfold higher using oleic acid compared to those from plain drug solution or carbopol gel. At the end of the skin permeation assay using ufasomes, up to 50% of the administered dose was found in the skin. These results suggest that methotrexate encapsulated in oleic acid vesicles may be of value for the topical administration of MTX in the treatment of psoriasis.

## 1. Introduction

Rheumatoid arthritis (RA) is a chronic inflammatory disease of unknown etiology and complex multifactorial pathogenesis. It is characterized by progressive and irreversible damage of the synovial-lined joints, resulting in the loss of joint space, bone, and a decrease in joint function and deformity [1]. RA is usually treated first with a nonsteroidal antiinflammatory drug (NSAID). However, current RA treatment favors early use of slow acting disease modifying anti-rheumatic drugs (DMARDs) because DMARDs have the potential to prevent or reduce joint damage. Therefore, they are used early in the treatment of RA and usually no later than 3 months after the commencement of NSAID treatment [2, 3]. Methotrexate (MTX) is one of the most frequently used DMARDs in the treatment of RA. Although the exact mechanism of action is still unclear, the efficacy of MTX is related to its cytotoxic and anti-inflammatory effects [4]. When administered in low weekly oral doses, MTX effectively suppresses inflammation in RA [5]. However, systemic toxicity effects such as stomatitis, nausea, bone marrowdepression, and liver toxicity can limit the oral use of this drug [6]. To reduce these effects, clinical studies have been done with topical methotrexate [7, 8]. A major problem in topical administration of methotrexate is that the drug is hydrosoluble and is mostly in the dissociated form at physiological pH: its capacity for passive diffusion is thus limited. One of the possibilities for increasing the penetration of drugs through the skin is the use of vesicular systems such as liposomes. Due to their biocompatibility and capability of incorporating both hydrophilic and lipophilic drugs, liposomes have recently been investigated as transdermal drug delivery systems [9]. The strategy of using liposomes is of interest but remains controversial owing to their large minimum size. To date, no consensus exists on whether or not administration of liposomes can lead to penetration into or through intact skin, but agreement is general that most liposomes do not reach deep into intact skin.

 In our previous study, we evaluated oleic acid vesicles as a carrier for sustained delivery of o 5-FU and zidovudine, Sharma et al., 2011 [10, 11], using topical/transdermal route. Therefore, aim of present investigation was to explore the potential of oleic acid vesicles as alternate for topical delivery of MTX.

## 2. Experimental

### 2.1. Preparation of Fatty Acid Vesicles

Oleic acid vesicles were prepared by film hydration method using Rota evaporator, as reported earlier by [12] with minor modification. Briefly, oleic acid of optimized molarity (80 mM), span80, and MTX was dissolved in methanol in a round bottom flask followed by evaporation of solvent under vacuum using a rotary evaporator (Roteva Equitron, Mumbai, India) (600 mm Hg, 100 rpm). For complete removal of any possible traces of methanol and also to prevent the formation of emulsion due to the residual organic solvent the completely dried film in rota evaporator was left overnight which was then hydrated at ambient temperature for 1 h with alkaline borate buffer (pH 7.4). The prepared vesicles were then sonicated to form the uniform size vesicular dispersion. Optimization and selection of carrier for topical delivery were performed by altering the ratios of oleic acid and MTX (Table 1). Unentrapped drug was separated from the vesicle dispersion by using gel chromatography on oleic acid vesicle presaturated Sephadex G-50 minicolumn using borate buffer as eluant. The encapsulation efficiency was determined by disrupting the eluted vesicles using sodium hydroxide solution (1 M) and subsequence estimation of released MTX, as entrapped drug.

### 2.2. Preparation of Plain MTX Gel

MTX containing carbopol gel was prepared by the method reported by Zao et al. (2007). Briefly, 1% w/v of carbopol 940 was dispersedinto purified water with the help of a vortex shaker (Tarsons, Kolkata, India) and allowed to hydrate for 4-5 h. The pH value of the gel was adjusted to 7.4 using triethanolamine. During preparation of the gel, to avoid any air entrapment, the solution was agitated slowly. Under gentle mechanical mixing for 5 min, plain drug gel was prepared by using an equivalent amount of MTX solution into the previously made carbopol gel in a 2 : 1 ratio.

### 2.3. pH Dependent Stability

Prepared vesicles were then incubated optimized with buffers of pH 8.5, 7.4, 6.5, and 5.5 to study the effect of pH on the stability. Drug release behavior was monitored by taking sample predetermined time intervals, the samples were withdrawn and centrifuged at 14,000 rpm for 30 min. The supernatant was analyzed for released free drug. The amount of drug leached was then calculated by the following formula:

(1) $$\text{\%Drug}\;\text{diffused}=\frac{\text{Amount}\;\text{of}\;\text{free}\;\text{drug}}{\text{Total}\;\text{drug}}\times 100.$$

Simultaneously, the incubated vesicles were observed for any change in morphology and size using an optical microscope. The studies were performed in triplicate.

### 2.4. Ufasomes Size Determination

The average diameter and size distribution of ufasomes suspensions were determined by photocorrelation spectroscopy using a 90 Plus Particle Size Analyzer (Brookhaven Instrument, NY, USA) at a fixed angle of 90° and at 25°C. Ufasomes suspensions were suitability diluted with phosphate buffer (pH 7.4) and filtered through a 1 m polycarbonate membrane to minimize interference particulate matter before sizing. Each measurement was in triplicate.

### 2.5. Entrapment Efficiency

The entrapment efficiency, calculated as percentage of initial MTX incorporated into liposomes, was determined after removing free MTX. Subsequently, ufasomes were redispersed in phosphate buffer (pH 7.4) containing 2% Triton X-100. The final clear solution was analyzed by HPLC for MTX content Determination of MTX release.

### 2.6. Differential Scanning Calorimetry (DSC)

DSC was performed with a Perkin-Elmer differential calorimeter (DSC7, Perkin-Elmer, Norwalk, CT, USA). Oleic acid (a), oleic acid—MTX vesicles (b), oleic acid—MTX 8 : 2 vesicles (c), and oleic acid—MTX 9 : 1 vesicles (d) were placed in conventional aluminum pan, and a scan speed of 2°C/min was employed. The weight of each sample was 12–15 mg.

### 2.7. The In Vitro Release of MTX Was Determined Using the Nonequilibrium Dialysis Method

 A locally fabricated cell system consisting of a donor and a receptor compartment of equal volume (1.5 mL) separated by a dialysis membrane (cutoff 12,000 Da) was used. Receiving medium was phosphate buffer (pH 7.4), and the cell was thermostated at 37°C. MTX aqueous solution and liposome suspensions were used as donor formulations. At fixed times, the receptor solution was tipped out and used for HPLC analysis and the cell was refilled with fresh phosphate buffer. The drug concentration was determined by HPLC.

The results were evaluated as apparent permeability constant of MTX (Kdapp cm min^−1^) calculated from the slope of the straight line obtained by plotting the amount of MTX diffused from the donor formulation versus time, assuming pseudo-zero-order kinetics.

### 2.8. Skin Irritation Studies

The oleic acid vesicle dispersions were tested for skin irritation as they have been reported to cause skin irritation owing to free acidic group present in the structure. The skin irritation test revealed negligible irritation scores in the case of oleic acid vesicle dispersion, while when the free oleic acid was applied, it produced erythematic events, as shown by the primary irritation scores (data not shown). The effect of formulations of the guinea pig skin after 24, 48, and 72 h of application was also visualized. It was found that oleic acid vesicles demonstrated skin tolerance of fatty acids may be because active groups of acid engaged in self-assembly, forming a noninvasive continuous membrane in the form of vesicles.

### 2.9. In Vitro Permeation and Skin Deposition Studies

Rat skin was used for permeation experiments using a vertical cell, as proposed by [13]. The hair of the outer skin surface was removed with dissecting scissors brought as close as possible to the skin without damaging it. The skin was carefully dissected with a scalpel and forceps. The skin was rinsed with normal saline and prehydrated by floating it with the stratum corneum upward on 0.002% w/v aqueous sodium azide to maintain an in vivo transepidermal hydration gradient [14]. The skin was then sandwiched between donar and receptor compartment with the stratum corneum side upwards. The receptor chamber was filled with 6 mL of buffer solution pH (7.4). The test formulation (1 mL) was transferred to donar compartment, which had an available diffusion area of 1.7 cm^2^ and left to dry. MTX aqueous solution was used a control formulation. The content of the receptor cell, continuously stirred at 37°C, was removed at appropriate intervals for HPLC determination, and the cell was immediately refilled with fresh receptor solution. At the end of the permeation experiments (24 h), the skin surface was washed five times with ethanol : buffer solution pH 7.4 (1 : 1) then with water to remove excess drug from the surface. The skin was then cut into small pieces. The tissue was further homogenized with ethanol : buffer solution pH 7.4 (1 : 1) and left for 6 h at room temperature. After shaking for 5 min and centrifuging for 5 min at 5000 rpm, the MTX content was determined by HPLC. Each experiment was repeated at least in triplicate from two different batches of the formulation.

### 2.10. HPLC Assay

The concentration of MTX was determined by HPLC. The HPLC system consisted of a pump (LC 20-AD), an UV detector (RF-551,  *λ* = 302 nm), a data station (Shimadzu, Kyoto, Japan), and a 5 cm C18 column (LiChrospher, Merck, Darmstadt, Germany). The mobile phase comprised methanol/acetonitrile/pH 5.4 buffer solution (8.5/6.5/85 v/v) and was delivered at a flow rate of 0.6 mL min^−1^. The injection volume was 20 L, and the relative retention time was found to be 9.8 min.

## 3. Stability Studies

 Stability of the product may be defined as the capability of a particular formulation to remain with the physical, chemical, therapeutic, and toxicological specifications. The optimized formulation (FAV-3) was selected for stability study on the basis of its in vitro performance and stored in tightly closed glass vials at room temperature and in refrigerator (4 ± 2°C). Following parameters were evaluated at different time intervals (20, 40, and 60 days). The formulations were stored in 10 mL glass vials at refrigeration temperature (4 ± 2°C) and room temperature for a period of 2 months. The samples were analyzed at predetermined time intervals visually and under optical microscope for the change in consistency and appearance of drug crystals. Vesicle size and size distribution were determined at definitive time intervals for a period of 2 months using stage eyepiece micrometer and haemocytometer, respectively, as described earlier.

## 4. Results

### 4.1. Fatty Acid Vesicle Characterization

**Figure 1:**
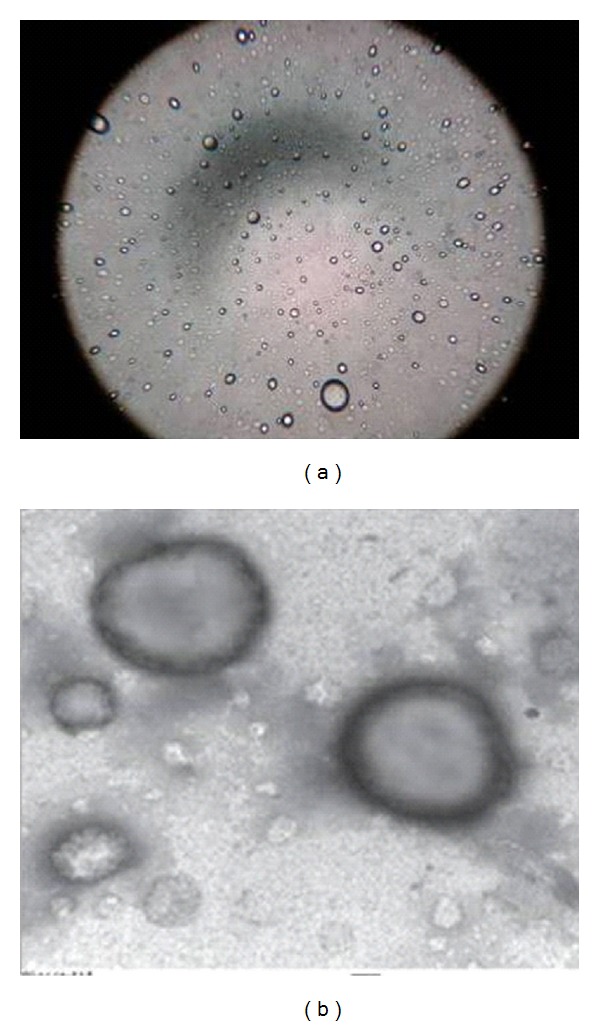
(a) Optical microscopy (400× magnification), (b) TEM photomicrograph of UF-3.

Multilamellar oleic acid vesicles were prepared by film hydration method by varying oleic acid-to-MTX molar ratios. Hydration was affected at room temperature for 1 h to enable complete hydration. The speed of rotation and concentration of oleic acid influence the thickness, uniformity, and duration of time interval of the film. The rotation speed of 100 rpm was observed to yield a uniform thin lipid film, while lower and higher rate, of rotation resulted in perceptible nonvesicular aggregated artifacts. The developed formulations were further characterized for size, PDI, and entrapment efficiency (Table 1). The studies carried out demonstrated no significant difference in the entrapment efficiency; however, the mean entrapment efficiency of the vesicles increased with an increase in the molar quantity of MTX up to 7 : 3 oleic acid to MTX ratio (51.0 ± 4.2%). Beyond this ratio, no further increase in drug entrapment was recorded. The vesicular sizes were obtained in the range of 500 nm to 1 *μ*m. The oleic acid vesicles dispersions obtained were mostly polydisperse; however, at 7 : 3 oleic acid-to-MTX ratio the dispersity was recorded to be 0.262 ± 0.037. The photomicrographs depict the spherical nature of the oleic acid vesicles (Figure 1(a)). Further, in addition, the TEM study conducted confirmed the ultrastructure of oleic acid vesicles which revealed multilamellarity of vesicles (Figure 1(b)).

### 4.2. Differential Scanning Calorimetry

**Figure 2:**
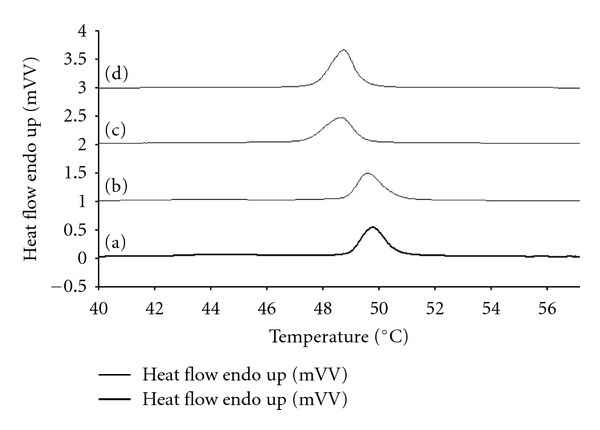
Differential scanning calorimetry traces of oleic acid (a), oleic acid—MTX vesicles (b), oleic acid—MTX 8 : 2 vesicles (c), and oleic acid—MTX 9 : 1 vesicles (d).

Differential scanning calorimetry was carried out to evaluate the interactions between MTX and oleic acid with different molar ratio; Figure 2 reports the thermograms. HPC was used for DSC measurements because its transition temperature (Tm) can easily be measured. The DSC trace of oleic acid vesicle showed a an enthalpy of 37.8 ± 0.4 J g^−1^ and peak transition at 48.9 ± 0.2°C. Incorporation of different molar ratio into oleic reduced the Tm value to 48.6 ± 0.2°C and the enthalpy to 35.7 ± 0.5 J g^−1^. The decrease in Tm value may indicate that the different molar perturbs the packing characteristics and, thus, fluidizes the lipid bilayer. The presence of MTX did not change either Tm or the enthalpy values, indicating that this molecule is entrapped in the hydrophilic core of oleic acid.

### 4.3. Drug Release Studies

#### 4.3.1. MTX Release

**Figure 3:**
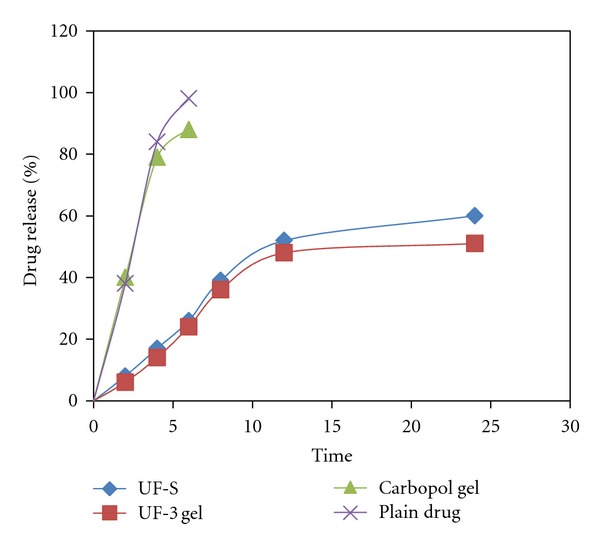
% drug release form different formulation across cellophane membrane.

The rate of release of MTX from ufasomal gel formulation was significantly lower (*P* < 0.05) than that from UF-3 gel formulation and drug solution (Figure 3). Maximum amount of MTX was released (97.2 ± 4.5%) within 4 hr from drug solution, while only 18.43 ± 1.5% and 16.38 ± 1.4% were released from UF-3 suspension and gel formulations, respectively. These results clearly indicated that the release of MTX gel was effectively retarded. For the characterization of the release kinetics, the in vitro drug release data was fitted to zero-order, first-order, and Higuchi equations [15]. Kinetic release parameters of different formulations are summarized in Table 3. The permeation studies revealed a zero-order release of MTX from ufasomal gel formulation (*r* ^2^ = 0.9961). Since theconcentration of drug is in equilibrium with the inner surface of the ufasomal vesicle membrane and diffusion path length is constant, therefore, zero-order permeation profile is generally expected with vesicular systems [16]. The release rate constant (log⁡*K*) for UF-3 gel, UF-3 gel suspension, and carbopol gel was found to be 0.7327, 0.8143, and 0.9923, respectively. The significantly (*P* < 0.05) lower  log⁡ *K*  value for MTX release from UF-3 gel as compared to UF-3 suspension and carbopol gel indicated that incorporation of oleic acid vesicles into carbopol gel further sustained its release.

#### 4.3.2. In Vitro Permeation and Skin Deposition

**Figure 4:**
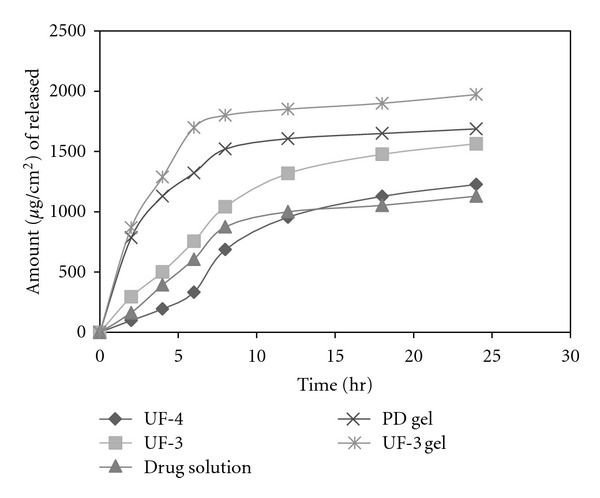
Amount of drug permeated from different formulation across rat skin for 24 hr.

The skin permeation study was conducted on optimized formulation prepared at a 7 : 3 fatty acid : drug ratio (pH 7.4) (highest entrapment efficiency and more uniform sized vesicles). To normalize the effect of pH on skin permeation, the plain pH of drug gel was also adjusted to pH 7.4. A significant increase in the skin permeation of MTX was recorded from oleic acid vesicle dispersion in comparison to plain gel (Figure 4). The amount of MTX permeated from the plain carbopol gel was 13 ± 3%. The drug penetration following application of an equivalent amount of drug in vesicular dispersion was significantly high, that is, 52 ± 6%. The permeation parameters were calculated by plotting a curve between cumulative amounts of drug permeated per unit area (*μ*g/cm^2^) versus time. The flux was obtained from the slope of the linear portion of the graph. The transdermal permeation rate constants obtained were higher for oleic acid vesicle dispersions (17 ± 1.4 *μ*g/h/cm^2^) than the plain drug gel (3.5 ± 0.7 *μ*g/h/cm^2^). It has also been observed that drug retained in the skin was more in the case of vesicular dispersion (29.55 ± 4.3) as compared to plain drug gel (5.06 ± 0.84%) (Table 2).

 During last decade, several studies have compared transdermal MTX transport from different vehicles, but the data are quite controversial; this may be due to several factors such as influence of the components on the skin barrier properties, on the different species and types of skin and on different experimental procedures used for study. In particular, [17] used 50% propylene glycol in aqueous medium at different pH and found a drug permeation parameters through human skin similar to that obtained from oleic acid vesicles, while [17, 18] reported very low MTX permeation from aqueous solutions. Other studies report MTX fluxes from vesicles [19]; the amount permeated through rat skin, calculated from the fluxes reported from UF-3 formulation, is similar to that now obtained from KG liposomes, while very low permeation 0 values were reported from other vesicular formulations [20]. However, to compare these last data, the possible enhancing effect of oleic acid must be considered. Table 3 also shows the residual amount of MTX in the skin after 24 h administration of different preparations. Surfactant with oleic acid incorporated promoted the transfer of MTX into rat skin. Skin deposition increased by a factor of 3 compared with either aqueous solution or carbopol gel.

In accordance with the results of our previous study Sharma et al. 2011 [21], where oleic acid vesicles applied nonocclusively, significantly improved in vitro skin delivery of 5-FU compared with aqueous solution. It is supposed that oleic acid can penetrate the skin whole, carrying the MTX entrapped in the hydrophilic core. Subsequently, a partition of intact vesicles into the deeper layers of the stratum corneum [22, 23] leads to high drug accumulation and only little vesicle materials probably reaches the deepest layers of the corneum and then the viable epidermis. However, other determinations, such as composition and vesicle size in donor and receiving medium, freeze-fracture electron microscopy or confocal laser scanning microscopy of the skin will be necessary to confirm this hypothesis. In conclusion, ufasomes obtained using oleic acid, a safe surfactant widely used in cosmetics, applied nonocclusively, improve in vitro skin delivery of MTX compared to either aqueous solution or normal liposomes. The enhanced accumulation of MTX within the skin might help to optimize targeting of this drug, creating new opportunities for well-controlled and modern topical application of MTX in the treatment of RA.

#### 4.3.3. pH-Dependent Stability

**Figure 5:**
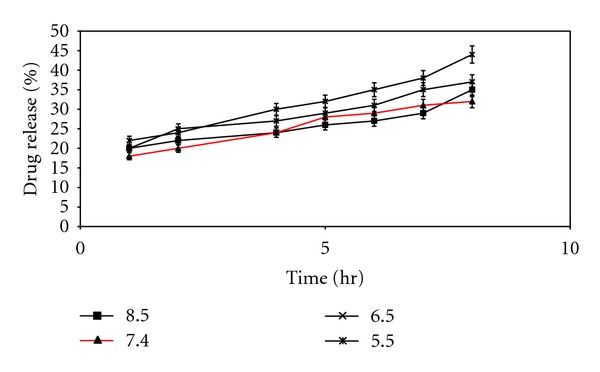
pH-dependent release behavior of drug from oleic acid vesicles dispersion.

**Figure 6:**
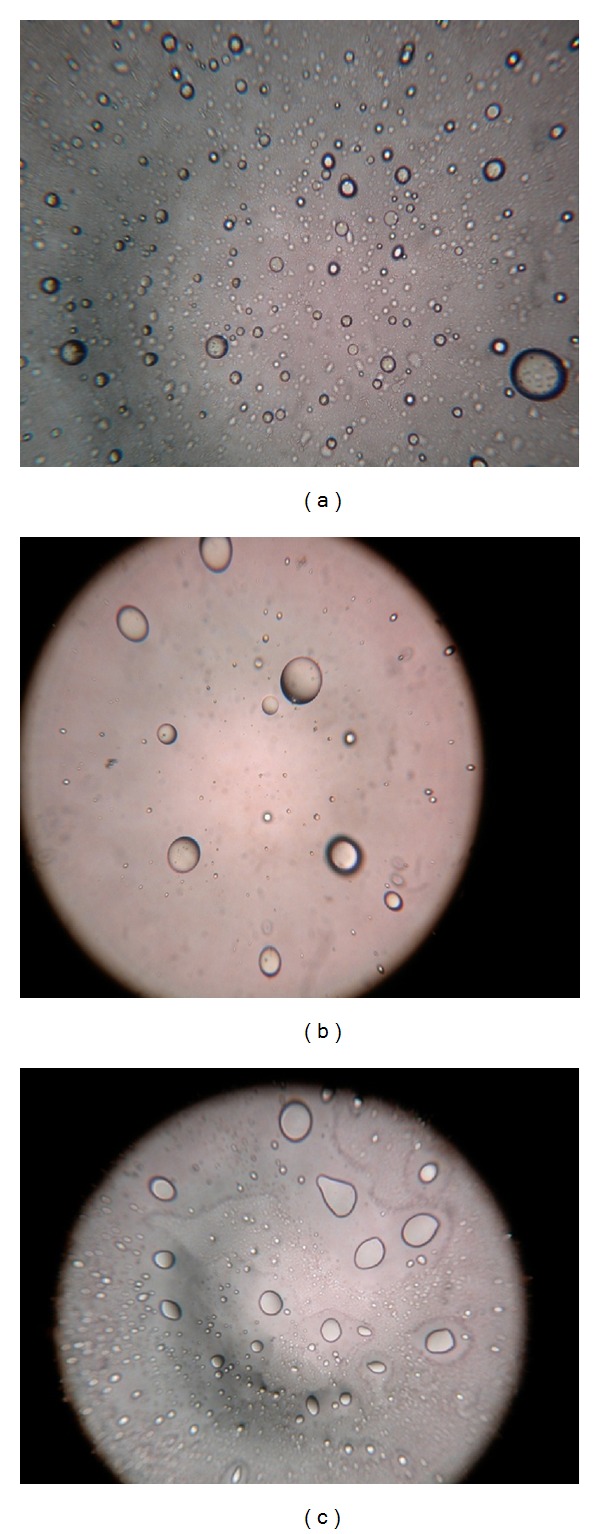
Photomicrograph of UF-3 formulation (a) pH (7.4), (b) pH (5.5), (c) pH (8.5).

 In order to understand, study to see the influence of pH dependent nature of the oleic acid vesicles was carried out as it provided useful information on topical drug delivery potential and characteristics of oleic acid vesicles since the pH of skin is 5.5. It was observed that the release from vesicles is highly pH-dependent and on lowering the pH from 8.5 to 5.5, only 20% of the drug remained in vesicles to be released after 8 h of incubation in buffer of pH 5.5 as compared to residual drug estimated, that is, 71% at pH 8.5 (Figure 5). The differences in drug diffusion recorded at pH 8.5 and 7.4 were not significant (*P* > 0.01). Therefore, further studies were continued by adjusting the pH of vesicles suspension to 7.4, since higher pH values may cause skin irritation and may not be acceptable for topical application. Simultaneously, morphological changes in vesicles size and shape were also observed with changing pH. The results displayed an increase in the size of the vesicles at low pH values (Figure 6).

## 5. Discussion

Critical factor for formation fatty acid vesicles that is, ufasomal formulation, is pH which controls the degree of ionization of fatty acid [24, 25]. Fatty acid (oleic acid) assembled into vesicles if pH equals the pKa of the acid (8.5), because, at this pH, *∼*50% of the carboxylic acid is ionized and transforms into ionized amphiphile(s) with a tendency to form vesicles aggregates. The acid is present as ionic RCOO^−^ as well as neutral RCOOH species. In such conditions, each ionized group is stabilized through a strong hydrogen bond formed with the neutral molecules. The negative charge present on the ionized carboxylic group is shared between two adjacent fatty acid molecules, that is, ionized and unionized and, thus, results in the formation of typical dimers. The hydrophobic hydrocarbon chain of so formed dimers protects itself from the aqueous compartment and thus orients to form an enclosed bilayer structure that minimizes the interaction between the hydrocarbon chain and water. The ratio of protonated and deprotonated group seems also critical in the process of vesiculation. This is possible only if the concentration of the fatty acid in aqueous dispersion exceeds the critical vesicle concentration (cvc), which is reportedly 80 mM for oleic acid [26]. The stability of the vesicles is attributed to the strong hydrogen bond-based interactions between the protonated and deprotonated groups, namely, RCOO^−^⋯ HOOCR as suggested and reported by [27–30]. Therefore, fatty acid vesicles were evaluated to assess their efficacy in delivering the bioactives to and through stratum corneum of the skin. The drug methotrexate (MTX) was used as a model drug.

In bid to understand the effect of drug : oleic acid ratio on the encapsulation study for MTX was carried out. It was observed that the drug bearing capacity of the oleic acid vesicles primarily depends upon the molar ratio of oleic acid and MTX. The entrapment efficiency increased up to oleic acid : drug molar ratio 7 : 3, beyond this ratio further increase in the amount of drug reduced the degree of drug encapsulation. As further addition of drug could have destabilized the vesicle membrane which in turn lead to the leakage of content due to the saturation of drug in the bilayer domain. Based on physicochemical characterization studied, UF-3 was selected for further studies. Further, by comparing the drug release data of oleic acid vesicles with that of MTX solution, it was concluded that the release of MTX from the vesicle was slow, controlled, and uniform as compared to plain drug solution. The pH-dependent stability behavior substantiates that drug diffusion across the skin may increase with a decrease in the pH of the vesicles dispersion. Thus, the increased diffusion of drug from the vesicles at low pH may have resulted due to decreased stability of the vesicles at lower pH. This further suggests that vesicles tend to fuse when they are exposed to low pH. This particularly holds for the pH that is lower than physiological pH. Moreover, the vesicles incubated in buffers of different pH were analyzed under an optical microscope. The optical images clearly revealed that the size of the vesicles slumped with the drop in the pH, proving the pH-dependent fusogenic tendency of vesicles.

## 6. Conclusion

In conclusion, ufasomes obtained using oleic acid, a safe surfactant widely, applied nonocclusively, improve in vitro skin delivery of MTX compared to either aqueous solution or normal liposomes. The enhanced accumulation of MTX within the skin might help to optimize targeting of this drug, creating new opportunities for well-controlled and modern topical application of MTX in the treatment of RA.

## Figures and Tables

**Table 1:** Size and entrapment efficiency of the prepared oleic acid vesicles.

Formulation code	Oleic acid: 5-MTX (molar ratio)	Entrapment efficiency	Particle size (nm)	PDI
UF-1	9 : 1	(39.4 ± 2.1)	505 ± 15	0.367 ± 0.037
UF-2	8 : 2	(45.4 ± 2.1)	523 ± 12	0.468 ± 0.037
UF-3	7 : 3	(51.0 ± 4.2%),	632 ± 17	0.262 ± 0.037
UF-4	6 : 4	(49.4 ± 2.7)	531 ± 16	0.489 ± 0.037
UF-5	5 : 5	(48.4 ± 2.4)	404 ± 13	0.581 ± 0.037

UF-1,2,3-ufasomes with different molar ratio of drug.

**Table 2:** % Cumulative amount of methotrexate permeated after 24 h (microgram MTX) and skin deposition (percentage MTX) from different vesicular formulations and from aqueous control solution.

	MTX (*μ*g)	MTX (% age)
Aqueous control solution	5.06 ± 0.84%	13 ± 3
carbopol gel	12.5 ± 2.8	30 ± 5
UF-3	21.5 ± 4.5	43 ± 4
UF gel	29.55 ± 4.3	52 ± 6
UF suspension	19.55 ± 4.6	45 ± 5

**Table 3:** Order of drug release of various formulations determined by the regression coefficients.

Formulation	**r^2^				^4∗^ *n*
	Zero order	First order	Higuchi	^3∗^Log *k *	
UF-3 gel	0.9961	0.8843	0.9792	0.7327	0.7574
UF-3	0.9742	0.7931	0.9534	0.8143	0.8304
Carbopol gel	0.8227	0.9964	0.9346	0.9923	0.9953
Plain drug	0.6081	0.9582	0.8687	1.2465	1.2314

^*∗∗*^Coefficient of correlation, ^3*∗*^kinetic constant, and ^4*∗*^diffusional exponent indicative of the mechanism of drug release.
